# Value of Coronary CT Angiography in Ruling Out Coronary Artery Disease in Elderly Patients Candidates to TAVI

**DOI:** 10.3390/jpm16050272

**Published:** 2026-05-19

**Authors:** Mattia Alexis Amico, Andrea Taddei, Matteo Casini, Carlo Fumagalli, Manlio Acquafresca, Mario Moroni, Angela Migliorini, Francesco Meucci, Carlo Di Mario, Niccolò Marchionni, Renato Valenti, Nazario Carrabba

**Affiliations:** 1 Cardio-Thoraco-Vascular Department, University Hospital Careggi, Largo Brambilla 3, 50134 Florence, Italy; 2 Department of Geriatric and Intensive Care Medicine, University Hospital Careggi, Largo Brambilla 3, 50134 Florence, Italy; 3 Department of Advanced Medical and Surgical Sciences, University of Campania Luigi Vanvitelli, 80138 Naples, Italy; 4 Department of Emergency Radiology, University Hospital Careggi, Largo Brambilla 3, 50134 Florence, Italy

**Keywords:** TAVI, cCTA, aortic stenosis, PCI

## Abstract

**Background**: Coronary computed tomography angiography (cCTA) is now indicated as a non-invasive tool for ruling out obstructive coronary artery disease (O-CAD) in patients who are candidates for transcatheter aortic valve implantation (TAVI) showing low-intermediate pre-test probability of O-CAD. In elderly and comorbid TAVI candidates, the safety and accuracy of cCTA as an alternative to invasive coronary angiography (ICA) for ruling out O-CAD remain to be established. **Aim**: To assess the feasibility, diagnostic accuracy, and clinical safety of cCTA for ruling out proximal O-CAD in elderly, comorbid, high-risk patients undergoing TAVI. **Methods**: We conducted a retrospective, single-center study including all consecutive patients with severe symptomatic aortic stenosis who underwent TAVI between January 2019 and December 2020. All patients underwent pre-TAVI cCTA. Patients with positive or non-diagnostic cCTA underwent ICA selectively (ICA group). In patients with no-O-CAD, ICA was omitted and proceeded directly to TAVI (no-ICA group). Accordingly, patients were divided into two groups: no-ICA and ICA group. Clinical follow-up was extended up to 5 years, with assessment of major adverse cardiovascular events (MACEs), mortality, heart failure hospitalizations, and unplanned revascularization. **Results**: Among 355 patients enrolled, 210 were included in the study. Among them, 140 (66.7%) had negative cCTA for O-CAD, and ICA was safely omitted in 132 patients (62.8%). cCTA was inconclusive in 43 patients (20.5%) and positive in 27 (12.9%). ICA confirmed O-CAD in 53 of 78 patients (67.9%) and PCI was performed in 35 of 53 (66.0%). The accuracy of cCTA for ruling in O-CAD was low (66.28%). During the follow-up period (1513 ± 508 days), the no-ICA group showed comparable outcomes to the ICA group in terms of periprocedural complications and long-term results—at both 1 and 5 years—for MACEs, heart failure hospitalizations, mortality and unplanned revascularization. Outcomes remain comparable between the two groups after performing matched-pair analyses. **Conclusions**: Our data show that cCTA may provide a reliable, safe, and effective alternative to ICA for ruling out obstructive CAD in elderly patients undergoing TAVI when image quality is diagnostic. A cCTA-based strategy allows deferral of ICA in most cases without compromising procedural safety or long-term clinical outcomes, enabling a personalized and tailored clinical pathway. Whether advanced CT techniques, such as CT-FFR and photon-counting CT, may help refine patient selection for invasive coronary assessment remains to be demonstrated.

## 1. Introduction

Computed tomography angiography (CTA) has become an essential tool in the pre-procedural assessment of patients undergoing transcatheter aortic valve implantation (TAVI). This imaging modality enables precise anatomical evaluation of the aortic valvular complex, root, ascending aorta, and peripheral vasculature, thereby contributing to improved procedural planning and outcomes [1,2,3].

Given the high prevalence of coronary artery disease (CAD) in the elderly population—frequently comorbid among TAVI candidates—coronary assessment remains a critical component of pre-TAVI evaluation. Traditionally, invasive coronary angiography (ICA) has been regarded as the reference standard for defining coronary anatomy, particularly considering the elevated pre-test probability of obstructive CAD (O-CAD) in this population [4]. Nevertheless, ICA is an invasive technique that carries the risk of bleeding, vascular complications, contrast-induced nephropathy, and increased costs (both procedural and hospitalization-related). In this setting, coronary CTA is considered a potential alternative for CAD assessment in patients undergoing valve interventions. It is estimated that 20–70% of candidates for preoperative evaluation can safely avoid ICA on the basis of coronary CTA results, thereby reducing procedure-related risks, radiation exposure, and resource utilization [1,2,3,5].

However, the role of CTA in coronary assessment has historically been limited by extensive calcifications, motion artifacts, and technical or physiological challenges, including the use of beta-blockers and nitrates, which may be poorly tolerated in patients with severe aortic stenosis (AS) [3,6].

With the progressive extension of TAVI indications to younger and lower-risk patients—who are less likely to harbor significant CAD—the potential of coronary CTA (cCTA) as a non-invasive gatekeeper for ruling out O-CAD has gained increasing interest. This approach could reduce unnecessary invasive angiography, limit procedural risks, radiation exposure, and healthcare costs, while maintaining diagnostic safety [2,4].

The 2025 ESC Guidelines for the Management of Valvular Heart Disease support the use of cCTA for rule-out CAD in TAVI candidates, reporting high sensitivity (95–97%) and moderate specificity (68–73%) for detecting obstructive CAD, mainly limited by coronary calcification and atrial fibrillation [4,7,8]. When cCTA is performed as part of standard pre-TAVI evaluation and provides images of adequate quality to rule out O-CAD, omission of invasive coronary angiography may be considered (Class IIa, Level B) [6,9,10].

Accordingly, the present study aims to evaluate the feasibility, diagnostic accuracy, and safety of cCTA for the detection of proximal O-CAD as a potential alternative to conventional ICA in candidates for TAVI.

## 2. Materials and Methods

### 2.1. Inclusion and Exclusion Criteria

We conducted a retrospective, observational, single-center study at Careggi University Hospital (Florence, Italy). The study protocol was approved by the Ethics Committee of Careggi University Hospital and complied with the principles of the Declaration of Helsinki, approval code CET ACEV 6267/EST/25, approval date 22 January 2026. Written informed consent was obtained from all participants.

All patients referring to Careggi University Huospital with severe symptomatic AS who underwent TAVI between 1 January 2019 and 31 December 2020 were considered. The decision for TAVI versus surgical aortic valve replacement (SAVR) was made by the institutional Heart Team, comprising general and interventional cardiologists, cardiothoracic surgeons, cardiac CTA specialists, cardiac anesthesiologists and geriatric specialists.

Exclusion criteria included: absence of informed consent; emergency procedures; unavailability of pre-TAVI cCTA; contraindications to cCTA (e.g., poorly controlled atrial fibrillation, hemodynamic instability, chronic kidney disease with eGFR < 30 mL/min/m^2^, or claustrophobia); Heart Team decision for SAVR; previous percutaneous coronary intervention (PCI) or coronary artery bypass graft (CABG) within the preceding 3 months; and previous PCI with stent diameter < 3 mm.

### 2.2. Study Protocol

Patients underwent a comprehensive echocardiographic assessment (pre-TAVI and pre-discharge) and were evaluated using the Multidimensional Prognostic Index (MPI), a composite score derived from ADL, IADL, nutritional status, comorbidities, cognition and mood, polypharmacy, and social support, to stratify procedural risk as low, intermediate, or high.

All patients underwent a cCTA to assess TAVI feasibility, select valve size, determine vascular access, and evaluate the presence of concomitant O-CAD. O-CAD was defined as >70% stenosis in the proximal left anterior descending artery (LAD), dominant right coronary artery (RCA), or dominant circumflex artery (LCX), or >50% stenosis in the left main (LM). Based on cCTA findings, patients without O-CAD proceeded directly to Heart Team evaluation, avoiding ICA. On the other hand, patients with evidence of O-CAD or inconclusive or non-diagnostic cCTA in proximal coronary segments were considered positive and underwent ICA. PCI was performed before or during TAVI procedure in patients with confirmed O-CAD suitable for revascularization. According to this workflow, patients were classified into two groups: the no-ICA group, in which ICA was omitted, and the ICA group, in which after cCTA patients underwent ICA prior to TAVI, including patients with O-CAD on cCTA and those with inconclusive or non-diagnostic cCTA results.

Clinical follow-up was conducted using hospital registries and scheduled outpatient visits. When clinical data was incomplete, additional information was obtained through direct telephone interviews with patients or their caregivers.

#### 2.2.1. cCTA Scan Protocol

A retrospectively ECG-gated CTA scan was performed alongside a high-pitch scan of the vascular access route, using a single intravenous bolus of 70 mL of iodinated contrast medium.

All CTA scans were conducted with a 128-slice dual-source CTA system (SOMATOM Definition Flash, Siemens Healthineers, Forchheim, Germany). The detector collimation was set at 2 × 64 detector rows × 0.6 mm, utilizing a flying focal spot technique and a gantry rotation time of 280 ms. Scout-based automatic tube current modulation (Care Dose 4D, Siemens Healthineers) was applied, with a reference tube current–time product of 320 mAs per rotation.

To achieve a target heart rate of ≤60 bpm, oral and/or intravenous beta-blockers or oral ivabradine were administered when necessary, but no nitrate was employed to avoid hypotension. A test bolus scan was used to calculate the contrast transit time: 15 mL of iodinated contrast medium followed by a 30 mL saline chaser was injected to measure the time to peak opacification in the proximal ascending aorta. This time, plus 2 s for the standard protocol or 5 s for the high-pitch protocol, was used to estimate the transit time. Subsequently, 65 mL of iodinated contrast medium, followed by a 50 mL saline chaser, was injected, with bolus tracking initiated when the ascending aorta region of interest (ROI) reached a threshold of 100 Hounsfield units above baseline attenuation.

In high-pitch spiral mode (flash mode), prospective ECG triggering was used to acquire a complete dataset within a single heartbeat, starting at 60% of the R–R interval. For the sequential mode (spiral technique), the center of the acquisition window was set at 70% of the R–R interval, covering the entire heart in three to four heartbeats with a step-and-shoot approach.

Coronary artery datasets were reconstructed with a 0.6 mm slice thickness, 0.4 mm increment, a 180 mm field of view, a medium-soft convolution kernel (B26), and a sharp convolution kernel (B46) in cases of significant coronary calcium. Reconstructed images were transferred to a dedicated workstation (MMWP, Siemens Healthineers) for evaluation. Axial images, multiplanar reformations, and maximum intensity projections were utilized to assess coronary arteries. In addition, Medixant RadiAnt DICOM Viewer software (Version 2021.1, 27 June 2021; https://www.radiantviewer.com) was employed for image analysis.

Coronary artery segments were classified following a modified American Heart Association protocol. Segments with a luminal diameter ≥ 1.5 mm were analyzed independently by two observers (N.C. and M.A., each with over 10 years of cCTA experience). Any discordances were resolved by a third observer (M.M., with over 8 years of cCTA experience).

Radiation dose was documented as the dose-length product (DLP) and effective dose (ED). The ED for each patient was calculated using the formula DLP × 0.014, applying a 0.014 mSv/Gy/cm conversion factor for chest radiation.

#### 2.2.2. Invasive Coronary Angiography

ICA and invasive physiology (fractional flow reserve [FFR]; instantaneous flow reserve [iFR]) was performed in accordance with the standard practice. A stenosis on ICA was defined as obstructive when any lumen reduction >70% was present. In patients with proximal stenosis of 50–80%, revascularization decision making (omitting RCA lesions in the event of left dominance) was guided by either FFR (threshold, ≤0.80) or iFR (threshold ≤ 0.89) measurements, while revascularization was performed without physiology-guidance when stenosis > 80%.

### 2.3. Endpoints

The primary endpoint was the difference in major adverse cardiovascular events (MACEs) between the two groups at 5-year follow-up. MACE was defined as a composite of all-cause mortality, non-fatal myocardial infarction or unplanned PCI, hospitalization for heart failure (HF), and hospitalization for any cardiovascular cause, including major arrhythmic events. Secondary endpoints included the evaluation of periprocedural complications within 30 days (including permanent pacemaker implantation, access-site bleeding or transfusion-requiring anemia, and contrast-induced nephropathy), and the analysis at 5 years of all-cause mortality and HF-related hospitalizations (both first and recurrent events). Differences in MACE and its main components were analyzed at 1- and 5-year follow-up.

### 2.4. Statistical Analysis

Continuous variables were expressed as mean ± standard deviation, and categorical variables as counts and percentages. The normality of data distribution was assessed using the Shapiro–Wilk test. Comparisons between groups were performed using Student’s *t*-test or Wilcoxon rank-sum test for continuous variables, and the chi-square or Fisher’s exact test for categorical variables, as appropriate. The cumulative incidence of clinical outcomes was estimated using the Kaplan–Meier method and compared with the log-rank test. As a sensitivity analysis, multivariable Cox proportional hazards regression models were fitted for all-cause mortality and major adverse cardiovascular events (MACEs), with ICA strategy as the main exposure variable (performed vs. not performed). Models were adjusted for age, sex, time to TAVI, glomerular filtration rate (GFR, assessed by Cockcroft–Gault), diabetes mellitus, and prior cardiovascular events. For the mortality analysis, follow-up time was calculated in days from TAVI to death or last available follow-up. For the MACE analysis, time-to-event was defined as days from TAVI to the first MACE, while patients without MACE were censored at the date of last available follow-up. Adjusted hazard ratios (HRs) with 95% confidence intervals (CIs) were reported. The proportional hazards assumption was assessed using Schoenfeld residuals. In addition, a propensity score matched analysis was performed to further reduce baseline imbalance between groups. Patients undergoing ICA were matched to patients in the no-ICA group according to age, sex, and GFR. Covariate balance after matching was assessed using standardized mean differences (SMDs), with values <0.10 considered indicative of adequate balance. Clinical outcomes in the matched cohort were then re-evaluated using time-to-event analyses. A two-sided *p* value < 0.05 was considered statistically significant. Primary analyses were performed using SPSS software (version 29.0; IBM Corp., Armonk, NY, USA), while survival, sensitivity, and matched analyses were performed using RStudio statistical software (R version 4.4.1; R Foundation for Statistical Computing, Vienna, Austria).

## 3. Results

### 3.1. Study Population

**Figure 1:**
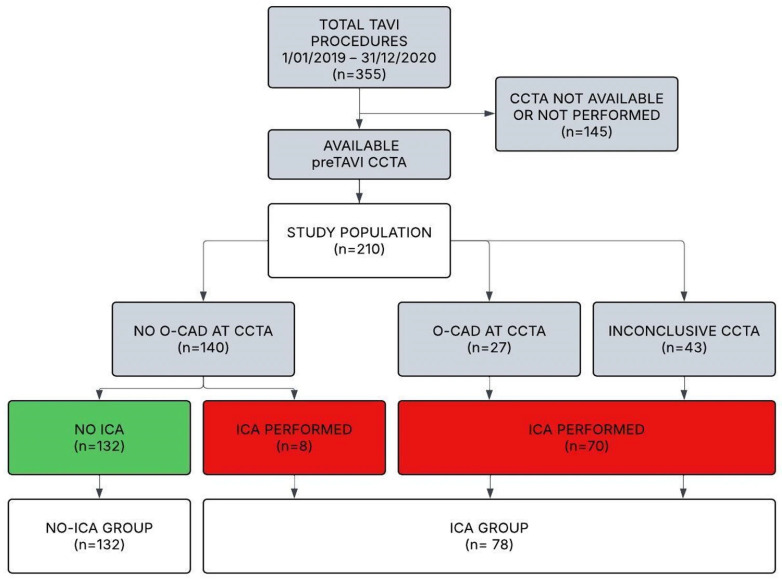
Flow diagram of patient selection and study groups. cCTA: coronary computed tomography angiography; ICA: invasive coronary angiography; O-CAD: obstructive coronary artery disease; PCI: percutaneous coronary intervention; TAVI: transcatheter aortic valve implantation.

During the study period, a total of 355 patients with severe AS were referred for pre-TAVI cCTA assessment. Of these, 210 patients met the study inclusion and exclusion criteria and were included in the final study cohort (Figure 1). Following cCTA, in 132 patients (62.8%) showing non-obstructive CAD, ICA was omitted, representing the no-ICA group. Conversely, ICA was performed in 78 patients (37.2%), constituting the ICA group. Among this group, 27 underwent ICA due to suspicion of significant proximal coronary stenosis, 43 due to inconclusive or non-diagnostic cCTA (10 caused by high-frequency artifacts and 33 by respiratory motion artifacts), and 8 at the discretion of the interventional cardiologist.

Baseline characteristics and medical history are summarized in Table 1. The mean age at TAVI was 83.6 ± 5.0 years, with no significant difference between the *no-ICA* and the *ICA* groups (83.5 ± 5.0 vs. 83.8 ± 5.2 years, *p* = 0.659). Female sex was more prevalent in the *no-ICA* group (68.2% vs. 38.7%, *p* = 0.005). Both groups had a high burden of comorbidities: more than 80% of patients had hypertension, and a relevant proportion had chronic kidney disease (eGFR < 60 mL/min/m^2^; 27.1% vs. 32.1%), without statistically significant differences between groups. No significant differences were observed between groups regarding body mass index (BMI), Euroscore II or STS score. MPI was significantly higher in the *no-ICA* group (*p* = 0.036).

Echocardiographic evaluation showed preserved left ventricle ejection fraction (LVEF) in both groups, which was slightly lower in the ICA group (52.1 ± 11.0% vs. 56.6 ± 10.0%, *p* = 0.004). No significant changes in LVEF were observed at post-procedural assessment. Pre- and post-procedural transvalvular gradients and aortic valve area did not differ significantly between groups. Overall, 31 patients (14.8%) had low-gradient AS, including 13 with true low-flow, low-gradient AS and 18 with paradoxical low-flow, low-gradient AS. At pre-discharge evaluation, approximately one-third of patients had a trivial/mild paravalvular leak (26.5% vs. 34.6%, *p* = 0.491). Echocardiographic findings are summarized in Table 2.

### 3.2. cCTA and ICA Findings and Subsequent Interventions

cCTA and ICA findings are summarized in Table 3 and Table 4, respectively. The mean total amount of contrast medium administered was higher in the ICA group than in the *no-ICA* group (144 ± 33 vs. 87 ± 14 mL; *p* < 0.0001), as was the mean cumulative radiation exposure (9.10 ± 3.14 vs. 6.4 ± 2.74 mSv; *p* < 0.001).

Of the 210 patients who underwent cCTA (100% of the study cohort), non-O-CAD was noted in 140 (66.7%). Among this subgroup, absence or minimal CAD was detected in 47 patients, and non-obstructive plaques (i.e., <70% stenosis) in 93 patients. Among these, despite no significant restenosis on cCTA, 8 patients underwent ICA at the discretion of the interventional cardiologist. Specifically, in four cases ICA was prompted by the presence of proximal coronary stents, which may limit the accuracy of cCTA due to blooming artifacts and adjacent calcification. In the remaining, other than the uncertainty of stent patency, ICA was performed in anticipation of self-expanding valve implantation, given the potential for more complex coronary access after TAVI compared with balloon-expandable devices.

In 43 patients (20.5%), cCTA was inconclusive or non-diagnostic due to respiratory motion artifacts (33 patients) or high-frequency artifacts (10 patients). In 27 patients (12.9%), cCTA showed O-CAD.

Of the 78 patients who underwent ICA (37.1% of the study cohort), O-CAD was confirmed in 53 patients (67.9% of those who underwent ICA), comprising 100% of patients with O-CAD on cCTA (27/27), 51.2% of patients with inconclusive or non-diagnostic cCTA results (22/43), and 50% of patients (4/8) with non-significant in-stent restenosis on cCTA.

Of the 53 patients with O-CAD at ICA, PCI was performed in 35 patients. Revascularization was not performed due to non-proximal lesion location (4 patients) or functionally non-significant stenosis on FFR/iFR evaluation (14 patients).

cCTA performance in ruling-in O-CAD in our cohort of TAVI patients was not optimal in comparison to ICA, demonstrating a sensitivity of 92.98% [95% CI 83.0 to 98.05%], specificity of 13.79% [95% CI 3.89 to 31.66%], positive predictive value of 67.95% [95% CI 64.32 to 71.37%], negative predictive value of 50.0% [95% CI 21.22 to 78.78%] and accuracy of 66.28% [95% CI 55.28 to 76.12%].

### 3.3. Clinical Outcome

The median interval between cCTA and TAVI was 68.0 ± 31 days, with no statistical differences between the *no-ICA* and the ICA group (72.0 ± 39.1 vs. 61.1 ± 37.9, respectively; *p* = 0.541). The mean length of hospital stay was 9.2 ± 6.3 days (8.7 ± 6.0 vs. 10.0 ± 6.7, *p* = 0.149). Procedural TAVI success was achieved in all cases, with no intraprocedural deaths or major complications such as cardiac arrest, myocardial rupture, conversion to SAVR, or need for extracorporeal membrane oxygenation (ECMO). During the procedure, inotropic support was required in six patients (three vs. three). The most frequent peri-procedural complications (within 30 days of the TAVI procedure) were atrioventricular block requiring permanent pacemaker implantation (*n* = 31; 20 vs. 11; *p* = 0.47) and bleeding at the vascular access site (*n* = 22; 10 vs. 12; *p* = 0.076). According to the BARC classification, 15 events were minor (BARC type 1–2: 7 vs. 8), whereas 7 were major (BARC type 3a, requiring transfusion: *n* = 6 [3 vs. 3]; BARC type 3b, requiring surgical revision: *n* = 1 [0 vs. 1]). No BARC type 3c or higher-grade bleeding events were observed. Major ischemic stroke occurred in two patients (one vs. one). Post-procedural acute kidney injury requiring continuous veno-venous hemofiltration (CVVH) occurred in five patients (two vs. three), all of whom had pre-existing renal impairment; two of these (both in the ICA group) progressed to permanent dialysis.

Clinical follow-up was available for all patients and extended over a five-year period, with a mean duration of 1513 ± 508 days (1523 ± 509 vs. 1495 ± 510, *p* = 0.703).

Overall, TAVI procedure was associated with good symptom relief in both groups with a significant improvement of NYHA class observed at 1-year (NYHA class I-II: 94% vs. 92%) and at 5-year in surviving patients (NYHA class I-II: 87% vs. 86%).

Considering the 1st-year follow-up, a total of 23 MACEs were recorded (24.2% of all events; 14 vs. 9), including 9 deaths (6 vs. 3), 11 heart failure hospitalizations (7 vs. 4), 1 major arrhythmic event (0 vs. 1), and 2 non-ST elevation myocardial infarction (NSTEMI) who underwent ICA without indication for revascularization (2 vs. 0).

Considering the 5-year follow-up, a total of 95 MACEs occurred (59 vs. 36, *p* = 0.838). The mean time to MACE was 831 ± 592 days (868 ± 615 vs. 771 ± 554, *p* = 0.431). During follow-up, 73 deaths were recorded (48 vs. 25, *p* = 0.526), with a mean time to death of 1077 ± 555 days (1058 ± 536 vs. 937 ± 536, *p* = 0.373). A total of 38 patients were hospitalized for cardiovascular causes (21 vs. 17, *p* = 0.287), including 23 for heart failure (14 vs. 9, *p* = 0.835). Overall, 32 hospitalizations for heart failure were reported, including first and subsequent events (19 vs. 13). A total of four non-fatal myocardial infarctions or unplanned revascularizations occurred (two vs. two), with a mean time of 974 days. Among these, one STEMI occurred in the ICA group (at 679 days from TAVI).

The adjusted Cox model including ICA, age, time to TAVI, sex, GFR, diabetes and prior events, showed that only sex and GFR were independent predictors of MACE (*p* = 0.005 for both) and all-cause mortality (*p* = 0.010 and *p* = 0.001, respectively) (see Table 5).

**Figure 2:**
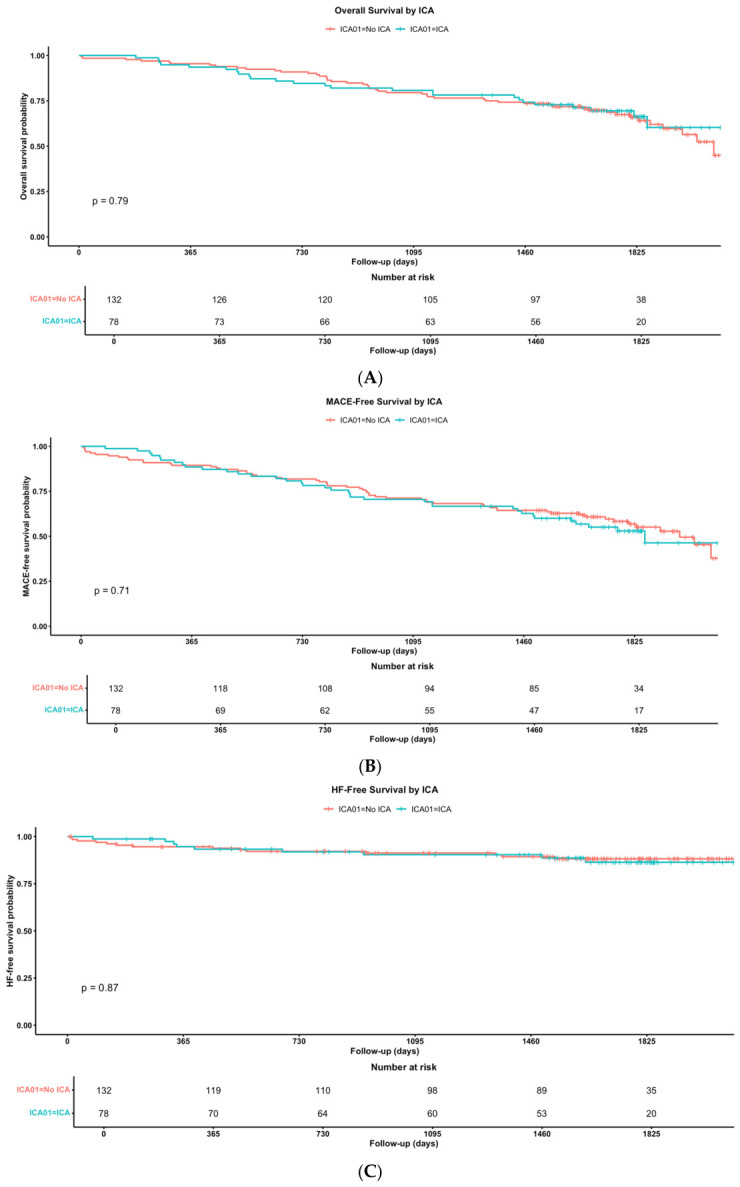
Five-year clinical outcomes according to ICA group. Kaplan–Meier curves for (**A**) MACE-free survival, (**B**), all-cause mortality and (**C**) heart failure hospitalization free survival.

Kaplan–Meier analyses for the 5-year follow-up are summarized in Figure 2. No difference was observed in event-free survival from MACE (Figure 2A). Similarly, single-endpoint analyses for all-cause mortality (Figure 2B) and for heart failure-related events (Figure 2C) also demonstrated comparable outcomes between groups.

**Figure 3:**
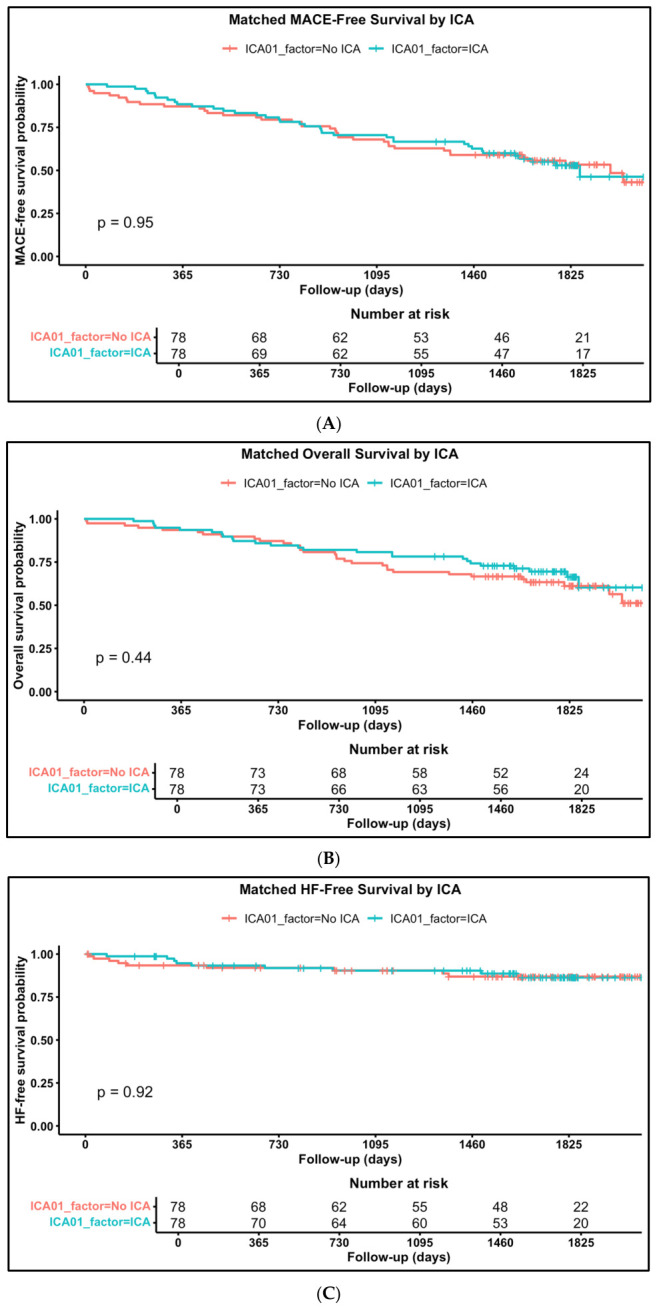
Propensity-matched score analyses, five-year clinical outcomes according to ICA group. Kaplan–Meier curves for (**A**) MACE-free survival, (**B**), all-cause mortality and (**C**) heart failure hospitalization-free survival.

After the proportional score matching between ICA (78 patients) and no-ICA (78 patients) group, Kaplan–Meier analyses for the 5-year follow-up confirmed that no difference was observed in event-free survival regarding MACE (Figure 3A), all-cause mortality (Figure 3B) and heart failure-related events (Figure 3C).

## 4. Discussion

Our study demonstrates that the systematic integration of cCTA into the pre-TAVI assessment is feasible and may be a safe strategy for rule-out O-CAD in patients with severe aortic stenosis. There are limited long-term outcome data and no randomized controlled trial evidence demonstrating the impact of CCTA-guided CAD management on MACE in TAVI patients. In our registry, an impressive 62.8% of planned ICA procedures were canceled in the pre-TAVI setting according to cCTA results, without any impact not only on procedural but also on long-term clinical outcomes.

Although ICA remains the mainstay of coronary assessment, procedural risks of ICA in TAVI patients, who are often old, frail and with multiple comorbidities, are not negligible [11]. Current European and American guidelines recommend revascularization to be considered in lesions located in proximal segments [12,13] due to a large area of myocardium at risk for severe ischemia [14]. Notably, symptoms in severe AS in most patients are caused by aortic valve disease and non-concomitant CAD. Thus, it is conceivable to test whether a combined CT strategy for both TAVI structural planning and proximal CAD assessment in a real-world setting would provide the information needed to perform TAVI. Following this strategy, ICA was safely canceled in more than half of patients. However, the diagnostic performance of cCTA, as a ruling-in tool, in our real-world population is not optimal and, in agreement with the prior literature, reflects the technical challenges posed by extensive coronary calcifications and higher heart rates causing blooming and motion artifacts in elderly TAVI candidates [15,16,17]. These findings remain a significant barrier to the universal adoption of a cCTA-based approach. However, continuous technological improvements, including higher temporal resolution, iterative reconstruction algorithms, motion correction techniques and the advent of more recent photo-counting CT are expected to enhance image quality and diagnostic confidence in forthcoming clinical practice. Notwithstanding the limitations of cCTA employed in our study, the absence of significant differences in clinical outcomes between the cCTA-only (no-ICA) and cCTA + ICA groups overall and in matched pair analyses reinforces the safety of deferring ICA in patients with minimal or non-O-CAD at cCTA even in a comorbid and elderly population. On the other hand, in revascularized patients, our findings confirm the low accuracy of cCTA in assessing the presence of in-stent restenosis in TAVI candidates, but also highlight the need to perform ICA before valve deployment according to the planned use of a self-expandable versus balloon-expandable valve, in order to avoid coronary access difficulties after the TAVI procedure in revascularized patients. The recent extension of indication for TAVI [13] in younger patients may enhance the role of cCTA in rule-out obstructive CAD, being less affected by extensive calcification of coronary arteries along with a greater ability to comply with breath-hold instructions by young patients.

### 4.1. Observational and Randomized Clinical Trials (RCTs) Studies

Observational studies provide data in agreement with our findings. A study by Snow et al. showed that CAD in an all-comer cohort of TAVI patients is an indicator of comorbidity and not associated with prognosis, and that most patients can be managed effectively with TAVI alone [18]. In a study by Abdel-Wahab et al., with comparable patients with coronary stenosis >50% undergoing TAVI alone vs. TAVI + PCI, patients had similar symptom relief and 6-month survival rates [19]. Chieffo et al. added to these studies by demonstrating in a real-world setting both feasibility and clinical relevance of using cCTA as an alternative to ICA in the TAVI diagnostic work-up [10]. Like our study, ICA was not performed based on the cCTA result in 70% of the patients with no negative clinical impact on 12-month clinical outcomes. The recently published CT2TAVI registry reinforces the feasibility of cCTA, particularly when combined with Computed Tomography Fractional Flow Reserve (CT-FFR), showing that ICA could be deferred in nearly 60% of TAVI patients without an increased rate of adverse events at 1 year [20]. Similarly, Becker et al. reported a cCTA sensitivity of 94.0% but only moderate specificity (72.4%) for ruling out obstructive CAD in TAVI patients, with specificity improving when CT-FFR was incorporated [21]. These findings highlight that, while cCTA is highly effective for excluding CAD, it is less reliable for confirming significant stenosis in heavily calcified arteries, and adjunctive technologies (CT-FFR, AI-based algorithms, photon-counting CT) may help mitigate this limitation in the future. In the ACTIVATION trial including patients with severe AS deemed suitable for TAVI and with ≥1 coronary stenosis ≥70% in a major epicardial artery (or ≥50% left main) relevant for PCI, there was no difference in rates of death and rehospitalization at 12-month follow-up, and PCI resulted in a higher incidence of bleeding [22]. A more recent NOTION-3 RCT [23] shows that adding PCI to TAVI reduces major adverse cardiac events (MACE) like heart attacks in patients with significant coronary artery disease (>90% stenosis in proximal LAD), making PCI a beneficial standard of care alongside TAVI for these complex cases. In our registry, no excess of periprocedural complications, in-hospital events, or long-term adverse outcomes was observed among patients managed without ICA. Rates of vascular complications, major bleeding (BARC 3a–3b), stroke, and acute kidney injury were comparable between groups, with no intraprocedural deaths, conversions to surgery, or need for mechanical circulatory support. Similarly, 1-year and 5-year event-free survival curves did not differ significantly, providing robust reassurance regarding the safety of a selective, cCTA-driven diagnostic strategy.

Notably, in our study among patients undergoing ICA after inconclusive or positive cCTA, approximately half were confirmed to have O-CAD, and one-third required percutaneous coronary intervention. These findings highlight the importance of integrating anatomical findings with clinical context and functional coronary assessment. Moreover, the symptom relief after the TAVI procedure was similar in both groups of patients, underscoring that in most patients, severe AS symptoms are caused by aortic valve disease and not by concomitant CAD. Whether cCTA with adjunctive techniques, such as CT-derived fractional flow reserve (CT-FFR), may further improve diagnostic accuracy, reduce false positives, and better stratify patients requiring invasive evaluation remains to be evaluated in dedicated studies.

### 4.2. CT-Guided Clinical Therapeutic Targeting

Our results suggest that cCTA may enable a selective strategy for coronary assessment in TAVI candidates, acting as a non-invasive gatekeeper to ICA. This approach could support risk-based allocation to ICA, potentially reducing unnecessary procedures while maintaining diagnostic safety. The integration of adjunctive tools (e.g., CT-FFR) may further refine patient selection, improving the precision of a personalized, CT-guided therapeutic pathway.

## 5. Limitations

This registry has several limitations. First, its retrospective, single-center, non-randomized design and moderate sample size may limit generalizability. Second, image acquisition and reconstruction parameters were operator- and device-dependent, and nitrates were not administered, potentially affecting coronary evaluation. Third, according to the study protocol, the analysis of diagnostic accuracy was confined to the subgroup of patients who underwent both cCTA and ICA, introducing possible selection bias. Fourth, advanced cCTA-based functional assessments, such as cCTA-FFR or plaque characterization, were not employed, which might have refined lesion assessment. Although promising as first steps, much remains to be learned about the value of cCTA-FFR in guiding the need for ICA in the TAVI work-up. Fifth, revascularized patients were included in our registry; however, diagnostic reliability in patients with coronary stents or bypass grafts is not well established, and diagnostic performance may vary according to the specific cCTA-FFR software, as well as the prevalence and severity of CAD. Moreover, the availability of cCTA-FFR is limited and may be costly. Finally, although the mean follow-up exceeded four years, the observational nature of the study precludes complete control of potential confounders. Despite these limitations, our registry, including revascularized patients, is truly representative of the real-world elderly TAVI population, but a cost-effectiveness analysis is essential to support the broader implementation of a cCTA-first strategy in preoperative pathways.

## 6. Conclusions

When image quality is diagnostic, cCTA may provide a reliable, safe, and effective alternative to ICA for ruling out obstructive CAD in elderly patients undergoing TAVI. A cCTA-based strategy allows deferral of ICA in most cases without compromising procedural safety or long-term clinical outcomes, enabling a personalized and tailored clinical pathway. Whether advanced CT techniques, such as CT-FFR and photon-counting CT, may help refine patient selection for invasive coronary assessment remains to be demonstrated.

## Figures and Tables

**Table 1:** Baseline characteristics and medical history.

	Study Cohort (*n* = 210)	No-ICA Group (*n* = 132)	ICA Group (*n* = 78)	*p*-Value
Age at implantation	83.6 ± 5.0	83.5 ± 5.0	83.8 ± 5.2	0.659
Female, *n* (%)	128 (61.0)	90 (68.2)	38 (38.7)	**0.005**
Hypertension, *n* (%)	175 (83.3)	111 (84.1)	64 (82.1)	0.758
Dyslipidemia, *n* (%)	105 (50.0)	64 (48.5)	41 (52.7)	0.576
Diabetes mellitus, *n* (%)	55 (26.2)	33 (25.0)	22 (28.2)	0.610
CKD, *n* (%)	57 (27.1)	32 (24.2)	25 (32.1)	0.219
Active smoking, *n* (%)	50 (23.8)	27 (20.5)	23 (29.5)	0.138
Atrial fibrillation, *n* (%)	49 (23.3)	33 (25.0)	16 (20.5)	0.458
BMI,	26.9 ± 4.6	25.7 ± 5.1	26.4 ± 4.7	0.358
Obesity, *n* (%)	34 (16.2)	20 (15.2)	14 (19.9)	0.157
Euroscore II	4.74 ± 4.00	4.59 ± 3.87	4.26 ± 3.87	0.188
STS score	3.37 ± 2.24	3.48 ± 2.66	3.42 ± 2.66	0.814
MPI	1.21 ± 1.26	1.46 ± 1.38	0.85 ± 0.99	0.036
Prior CV disease, *n* (%)	152 (72.4)	92 (69.7)	60 (76.9)	0.258

BMI: body mass index; CKD: chronic kidney disease (eGFR < 60 mL/min/1.73 m^2^); CV: (eGFR < 60 mL/min/1.73 m^2^); EuroSCORE II: European System for Cardiac Operative Risk Evaluation II; ICA: invasive coronary angiography; MPI: (eGFR < 60 mL/min/1.73 m^2^); and STS score: Society of Thoracic Surgeons score.

**Table 2:** Echocardiographic findings.

	Study Cohort (*n* = 210)	No-ICA Group (*n* = 132)	ICA Group (*n* = 78)	*p*-Value
Baseline LVEF, %	54.9 ± 10.6	56.6 ± 10.0	52.1 ± 11.0	**0.004**
Baseline peak aortic gradient, mmHg	78.1 ± 17.7	79.3 ± 19.1	75.8 ± 14.7	0.205
Baseline mean aortic gradient, mmHg	48.3 ± 11.2	49.5 ± 11.9	46.0 ± 9.3	0.047
Baseline AVA, cmq	0.69 ± 0.17	0.70 ± 0.18	0.67 ± 0.15	0.407
Post-procedural LVEF, %	55.8 ± 10.1	57.6 ± 9.6	52.7 ± 10.1	**0.004**
Post-procedural peak aortic gradient, mmHg	16.2 ± 7.7	15.7 ± 7.2	17.1 ± 8.6	0.275
Post-procedural mean aortic gradient, mmHg	9.2 ± 4.5	9.0 ± 4.4	9.6 ± 4.7	0.576
Paravalvular leak, *n* (%)	62 (29.5)	35 (26.5)	27 (34.6)	0.491

AVA: aortic valve area; LVEF: left ventricle ejection fraction; ICA: invasive coronary angiography.

**Table 3:** cCTA findings.

	Study Population (*n* = 210)
Normal coronary artery/No significant CAD, *n* (%)	39 (18.6)
Proceeding to ICA for proximal coronary stent, *n* (%)	8 (3.8)
Non-evaluable, high-frequency artifacts, *n* (%)	10 (4.8)
Non-evaluable, respiratory artifacts, *n* (%)	33 (15.7)
Non-obstructive CAD (<70% stenosis), *n* (%)	93 (44.2)
Obstructive CAD (>70% stenosis), *n* (%)	27 (12.9)
Single-vessel disease, *n* (%)	17 (8.1)
Double-vessel disease (including bifurcation), *n* (%)	10 (4.8)
Triple-vessel disease, *n* (%)	0 (0)

CAD: coronary artery disease; cCTA: coronary computer tomography angiography; and ICA: invasive coronary angiography.

**Table 4:** ICA findings.

	ICA Group (*n* = 78)	Clinical Reason (*n* = 8)	O-CAD at cCTA (*n* = 27)	Inconclusive cCTA (*n* = 43)
Absence/minimal CAD, *n* (%)	15 (19.2)	2 (25)	0 (0)	13 (30.2)
Non-obstructive CAD, *n* (%)	10 (12.8)	2 (25)	0 (0)	8 (18.6)
Obstructive CAD, *n* (%)	53 (52,6)	4 (50)	27 (100)	22 (51.1)
Single-vessel disease, *n* (%)	29 (37.2)	2 (25)	12 (44.4)	15 (34.9)
Double-vessel disease (including bifurcation), *n* (%)	24 (30.8)	2 (25)	15 (55.6)	7 (16.2)

**CAD**: coronary artery disease; **cCTA:** coronary computer tomography angiography; **ICA**: invasive coronary angiography; and **O-CAD**: obstructive coronary artery disease.

**Table 5:** Sensitivity analysis: adjusted Cox regression models for all-cause mortality (overall survival) and major adverse cardiovascular events (MACEs).

	Overall Survival			MACE		
	HR	CI	*p*-Value	HR	CI	*p*-Value
ICA (vs. no-ICA)	0.72	0.43–1.20	0.202	0.87	0.56–1.35	0.536
Age	1.03	0.98–1.09	0.190	1.03	0.99–1.08	0.192
Time to TAVI	1.00	1.00–1.00	0.899	1.00	1.00–1.00	0.984
Sex	0.48	0.27–0.84	0.010	0.51	0.31–0.82	**0.005**
GFR	0.97	0.95–0.99	0.001	0.98	0.96–0.99	**0.005**
Diabetes	1.45	0.85–2.47	0.177	1.37	0.85–2.19	0.196
Prior CV disease	0.83	0.48–1.41	0.487	0.85	0.53–1.36	0.496

CI: confidence interval; GFR: glomerular filtration rate; HR: hazard ratio; ICA: invasive coronary angiography; MACE: major adverse cardiovascular events; and TAVI: transcatheter aortic valve implantation.

## Data Availability

The data supporting the findings of this study are not publicly available due to privacy and ethical restrictions.
